# Isothermal and Thermomechanical Fatigue Behavior of 316LN Stainless Steel Under Torsional Loading

**DOI:** 10.3390/ma18030541

**Published:** 2025-01-24

**Authors:** Yiming Zheng, Fang Wang, Caijun Xu

**Affiliations:** Fujian Boiler and Pressure Vessel Inspection and Research Institute, Fuzhou 350008, China; z10zym@tju.edu.cn (Y.Z.); fjtjxucaijun@163.com (C.X.)

**Keywords:** 316LN, torsional loading, thermomechanical fatigue, isothermal fatigue

## Abstract

316LN austenitic stainless steel is extensively utilized within the domain of nuclear power, where its susceptibility to high-temperature fatigue and thermomechanical fatigue has emerged as a pivotal area of research for this material. Nevertheless, prior investigations have predominantly concentrated on axial loading outcomes, with a notable absence of studies examining its fatigue failure behavior under torsional loading conditions. The present study undertakes isothermal fatigue testing at temperatures of 450 °C, 550 °C, and 650 °C, along with thermomechanical fatigue testing across a temperature range of 350–550 °C, with strain amplitudes of 0.6%, 0.8%, and 1.2%. The findings reveal that secondary hardening observed under conditions of small deformation is primarily attributed to the enhancement of frictional stress, stemming from the accumulation of planar slip. Furthermore, as the temperature escalates, variations are observed in the intensity of the dynamic strain aging and the dislocation density within the material.

## 1. Introduction

Pressurized water reactor (PWR) primary pipelines plays an important role in nuclear power plants, and are key structure and safety barriers in nuclear power plants. The normal operation of the primary pipeline is an important guarantee for the smooth working of the nuclear power plant. Austenitic stainless steel, such as 304L, 316L(N), is often used as the structural material for the piping of nuclear power plants because of its excellent mechanical properties and high temperature corrosion resistance [1,2,3,4]. During its service, a series of thermal transients, such as thermal stratification, fluctuations, and turbulence due to the start-up and shutdown of the nuclear power plant and the internal flow, as well as rheological vibrations and changes in pressure loads, also lead to cyclic mechanical loads in the piping, which subject the material to the combined effect of alternating temperatures and mechanical loads. Failure under the combined effects of cyclic mechanical loading and temperature is defined as thermomechanical fatigue (TMF) failure, which has become an important direction in the study of nuclear pipeline failure [5,6,7,8]. The life design method current commonly applied is to use the life of the material at the highest temperature in the expected service condition under constant temperature, namely isothermal fatigue (IF) conditions, to evaluate the performance of the material in TMF conditions. However, due to the complex mechanisms of materials under TMF conditions, this approach may be unreasonable, and non-conservative results have been found in some reports [9,10]. Therefore, an in-depth study of the TMF behavior of austenitic stainless steel materials, as well as the intrinsic mechanisms, is of far-reaching significance for the evaluation of nuclear power safety, as well as the extension of service life.

As a kind of austenitic stainless steel with excellent performance, 316LN is widely utilized in third-generation PWR nuclear power plants due to its high strength, high creep resistance, and high resistance to high temperature corrosion [7,8,9,10,11]. Therefore, it is necessary to discuss the TMF properties of 316LN materials. In recent years, a series of studies have been carried out on the TMF properties of 316LN stainless steel. The current relevant studies mainly focus on the discussion of TMF properties of 316LN under axial loading. The temperature range and strain amplitude are the most concerned elements in TMF studies [12,13]. Nagesha et al. [5,14] evaluated the behavior of TMF in different temperature ranges. Reddy et al. [6,15] discussed the TMF and IF-T_max_ behavior of 316LN stainless steel with different nitrogen contents (0.07, 0.14, and 0.22 wt%) and different strain amplitudes (0.25%, 0.4%, 0.6%, and 0.8%). There are also more studies on dynamic strain aging and its potential mechanisms as one of the most important deformation mechanisms of 316LN materials at elevated temperatures [16,17,18]. On the basis of these studies, the study of TMF life and crack growth rate has also received attention. A series of effective TMF life prediction models provide scientific support for the fatigue design and safety assessment of high-temperature service components. Zheng et al. [19,20] carried out a study on the crack extension behavior of 316LN stainless steel under TMF loading conditions and discussed the effects of different loading forms on the crack extension rate. In addition, some studies have been carried out to investigate the fatigue performance of 316LN materials obtained through additive manufacturing at elevated temperatures, and the effects of heat treatment processes on fatigue life and mechanical properties are discussed [21].

Although there have been relatively abundant studies on the TMF behavior of 316LN materials, most of these studies have focused on the failure behavior of the materials under axial loading. Considering the complexity of the stress field and structural geometry, 316LN stainless steel is usually subjected to more complex torsional or even multi-axial stress states during service. However, the number of studies on the TMF failure behavior of 316LN under torsional loading or multiaxial loading conditions is more limited. Li et al. [22] discussed the differences in the failure behavior of 316LN under different equivalent shear strain amplitudes in the range of 250–450 °C, and Lin et al. [23] also carried out multiaxial fatigue tests of 316LN under IF and TMF conditions and found that the multiaxial non-proportional loading caused much more damage than uniaxial loading. The results of multiaxial TMF tests on high-temperature alloys [24,25,26] and TiAl alloys [27] also showed significant differences in cyclic deformation behavior and fatigue life between TMF tests with different loading directions. Other engineering materials have similarly demonstrated that the introduction of torsional loading will substantially increase the accumulation of fatigue damage and accelerate specimen failure [28,29,30,31,32,33]. These studies indicate that only conducting thermo-mechanical fatigue cracking tests under axial loading is not sufficient to provide adequate theoretical guidance to engineering applications, and the failure behavior of materials under torsional loading is of great significance for engineering vehicle applications. Therefore, IF and TMF tests with cyclic torsional loading are required to provide valuable information for structural design and structural integrity assessments of pipelines in nuclear power plants.

This study focus on the difference between variable temperature conditions (ranging from 450 °C to 650 °C) under torsional loading of 316LN material, and we mainly perform different IF tests and TMF tests with different strain amplitudes at 350~550 °C.

## 2. Materials and Methods

### 2.1. Specimen

**Figure 1:**
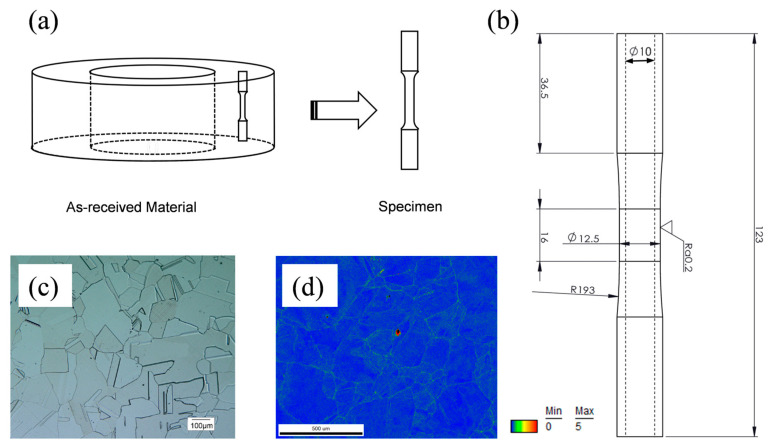
(**a**) Specimens were taken along axial direction of the 316LN pipeline. (**b**) Detail size of the specimens. (**c**) Metallographic picture of the as-received material. (**d**) KAM mapping result of the as-received material.

All torsional tests in this study used the thin-walled tubular specimens taken along the axial direction of the 316LN pipe wall, their sampling schematic is shown in Figure 1a. Figure 1b illustrates the detail size of the specimen under torsional test, which is designed according to the standard of ASTM E 2207 [34]. Figure 1c is the as-received material of grinding and polishing after electrolytic corrosion to obtain the metallographic picture, it can be seen that the material as a whole is the diameter of the equiaxed grains. The material has been heat-treated to relieve stress. By analyzing the kernel average misorientation (KAM) mapping result of the base material in Figure 1d, it can be seen that the as-received material has uniform grain size, equiaxial distribution, a grain diameter of about 127 μm (range from 103 μm to 162 μm), and no obvious residual stress. The specific compositions of the materials are obtained by energy dispersive spectrometer (EDS) mapping, as shown in Table 1. The specimen surfaces were well polished prior to the start of the test to ensure that there were no obvious scratches on the specimen surfaces, allowing for the specimen surfaces to be directly characterized after testing.

### 2.2. Test Details

Strain control tests were performed using an MTS 370.02 (MTS, Eden Prairie, MN, USA) multi-axis electro-hydraulic servo tester and an Epsilon 7650 (Epsilon, Irvine, CA, USA) high temperature multi-axis extensometer. Type K thermocouples with a diameter of 0.25 mm were spot welded in the middle of the scale length for dynamic temperature measurement and control. The medium frequency power supply (to avoid the skin effect) together with the induction coil can meet the fast heating rate (in this study, the heating rate is 3.33 °C/s). To ensure the accuracy of the temperature control in the TMF test, especially in the cooling section, two symmetrical air nozzles were used to assist the cooling operation, and the air source was supplied by a 0.2 MPa air compressor. More details about the experimental platform can be obtained from Zheng’s previous TMF studies [35,36].

**Figure 2:**
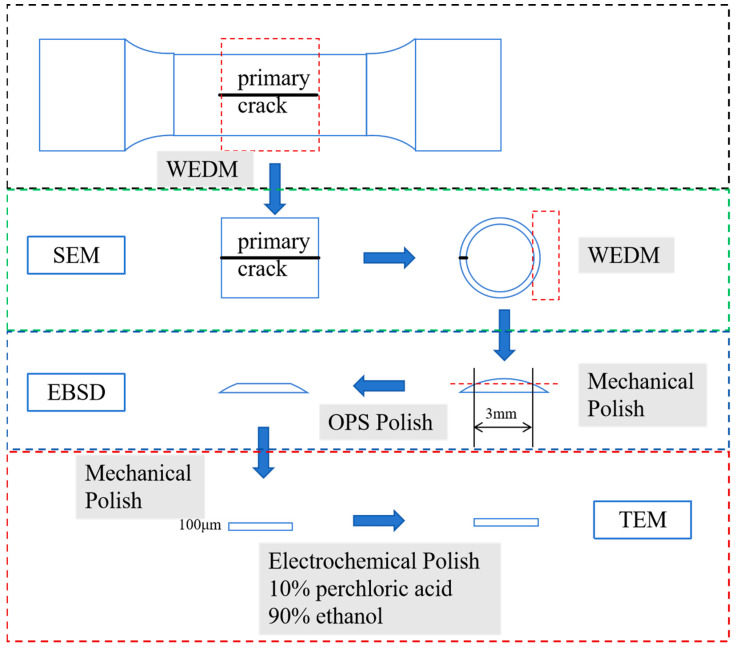
Schematic diagram of specimen processing and microscopic characterization process.

After the tests, the specimens were treated by wire-cut electrical discharge machining (WEDM) and polishing, after which the specimens were subjected to various types of advanced microscopic characterization. Scanning electron microscopes (SEM, Hitachi-S4800, Tokyo, Japan) were used to characterize the surface morphology and fracture morphology, and oxidation behaviors of the specimen surfaces were discussed. Specimens were polished using sandpaper and Al_2_O_3_ oxide polishing suspension (OPS). Then, electron back scatter diffraction (EBSD, TEAM™ Pegasus, EDAX, Pleasanton, CA, USA) was used to determine the crystal orientation and local dislocation distribution, with shooting parameters of 26 kV with 2.6 nA. In addition, dislocation sub-structures were observed using a transmission electron microscope (TEM, FEI Tecnai G2-F30, Hillsboro, OR, USA) operating at 300 kV. The flakes used for TEM observation were mechanically polished using progressively increasing amounts of sandpaper with abrasive paste until the thickness was 100 μm. Afterwards, electrolytic polishing was carried out in a perchloric acid-ethanol solution at a concentration of 10% at 35 V and −30 °C. The samples used for EBSD and TEM characterization were sectioned parallel to the sample axis, as shown in Figure 2.

The tests performed in this study included IF tests and TMF tests. All tests were strain controlled with each strain and temperature cycle fixed at 120 s. It is important to note that the strains discussed in this study are equivalent shear strains based on the von Mises criterion, which facilitates comparisons with axial tests in subsequent studies. Specifically,

(1) $$\gamma_{eq}=\frac{\sqrt{3}}{3}\times \gamma_{a}$$

(2) $$\tau_{eq}=\sqrt{3}\times \tau_{a}$$

where *γ_eq_* and *τ_eq_* were the equivalent shear strain amplitude and corresponding equivalent shear stress amplitude, respectively. *γ_a_* and *τ_a_* were the shear strain amplitude applied to the specimen and corresponding shear stress amplitude, respectively.

**Figure 3:**
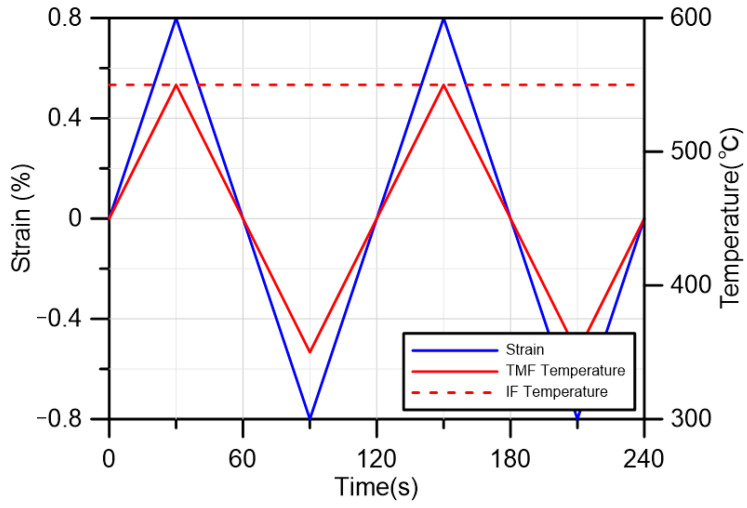
Schematic diagram of IF test and TMF test.

The control diagram for the test is shown in Figure 3. The IF test was conducted under three conditions of 450 °C, 550 °C, and 650 °C, with strain amplitudes of 1.2%. The temperature range of the TMF test is 350~550 °C, and three strain amplitudes of 0.6%, 0.8%, and 1.2% were conducted. The test temperatures were selected mainly in consideration of the load bearing temperatures of the 316LN material under actual service conditions. The loading rate and amplitude were selected mainly for comparison with other previous axial loading tests [35,36]. For torsion tests, the positive and negative values of strain and stress only represent the direction of strain and stress. Therefore, for all tests, the direction of loading at the beginning of the experiment is defined as the positive direction, and the direction of stress generated is the positive stress direction.

## 3. Results and Discussion

### 3.1. Cyclic Stress Response

**Figure 4:**
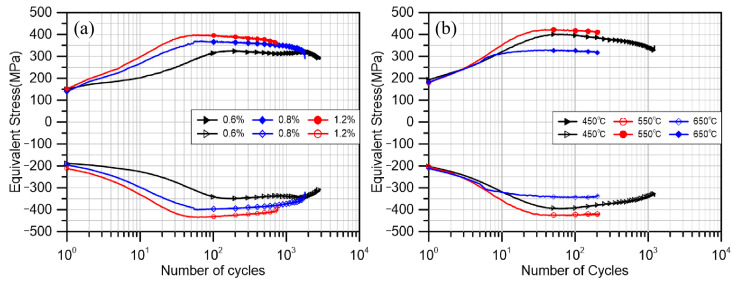
(**a**) Equivalent stress curve of TMF. (**b**) Equivalent stress curve of IF tests.

All experiments were stopped after significant cracks (3 mm or more) appeared and the cracks did not occur at the thermocouple welding spot. Figure 4 shows the results of all experiments. As shown in Figure 4, most experiments follow the process of cyclic hardening, followed by cyclic softening, and finally by failure. At a strain amplitude of 0.6% in the thermal test, a secondary hardening phenomenon appeared in the cyclic stress, which had also been reported in previous thermal fatigue studies of the material and explained as the result of plane slip under small deformation values [37]. Through the results of the constant temperature test, it can be clearly seen that with the same load of 1.2%, the test life at 550 °C and 650 °C is very close, but the cyclic stress of the former is significantly higher than that of the latter, even higher than the test at lower temperatures (450 °C).

This anomaly in the stress response at high temperatures is usually caused by the phenomenon of dynamic strain aging (DSA). The essence of the DSA phenomenon is the interaction of movable dislocations with interstitial or displaced solid solution atoms during plastic deformation, i.e., the solid solution and dislocations are pinned to and detached from each other during cyclic deformation [38,39]. It usually manifests itself as a serration in the hysteretic loop curve. An active DSA often implies a higher stress response. This is because the solid solution atoms and dislocations impede the movement of the dislocations when they are pinned to each other, which means that the deformation behavior of the material is internally resisted at this point, and higher loading conditions are required in order to complete the deformation [40,41]. In addition, the large amount of DSA behavior means that the dislocation density inside the material is high, which contributes significantly to cyclic hardening [42,43,44,45].

**Figure 5:**
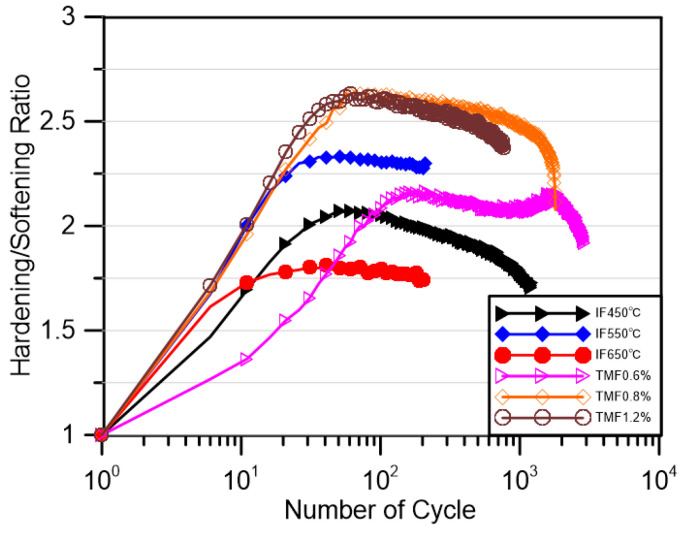
Hardening/softening ration of IF and TMF tests.

The hardening/softening relationship of the material during cyclic deformation was calculated as shown in Figure 5, where the peak stress of each cycle was divided by the peak stress of the first cycle of the test to obtain the hardening/softening rate of each test over the full life course. As can be seen in Figure 5, the hardening rate in the hardening phase of the IF test at 550 °C is significantly higher and similar to that of the TMF test.

The strength of DSA is closely related to the test conditions, especially the test temperature. In a series of previous tests on 316LN, it has been shown that under axial loading, the performance of DSA at 550 °C is significantly stronger than that at other temperatures [46]. In the present study, it can be concluded that under torsional loading and the same strain rate, 550 °C is still the most active temperature for 316LN.

The IF test specimens also showed a high hardening rate at 650 °C. This indicates that the DSA phenomenon of considerable intensity also occurs at 650 °C at the beginning of the experiment. However, the hardening phase at 650 °C is significantly shorter than that of the two sets of tests at lower temperatures, and after a short cyclic hardening period, the material enters a smooth cyclic softening period until final fracture. The results indicate that two deformation mechanisms, cyclic hardening and cyclic softening, compete with cyclic loading at the same time at this temperature, and the cyclic softening mechanism rapidly dominates in this test.

**Figure 6:**
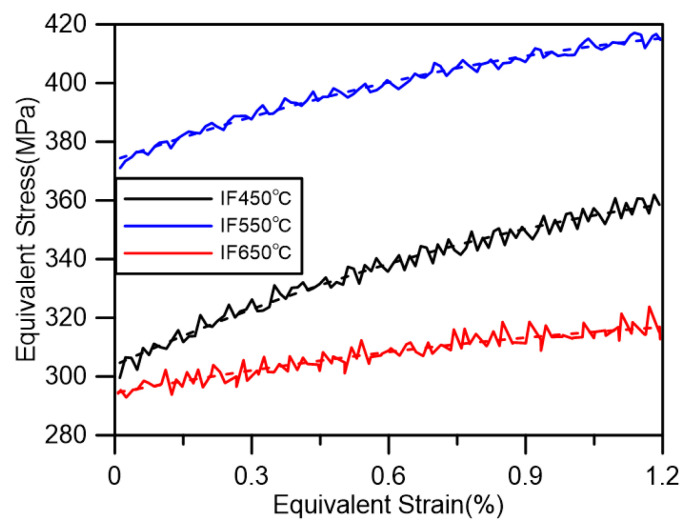
The serration feature in the half-life hysteresis loop of IF tests at different temperatures.

The serration feature observed on the hysteresis loop is indeed a macroscopic indicator of dynamic strain aging (DSA). This phenomenon manifests as jagged undulations on the stress–strain curve, reflecting the intricate interaction between movable dislocations and solid solution atoms. When numerous movable dislocations become pinned by solid solution atoms, they are unable to continue their movement. This leads to an increase in the material’s resistance to deformation, resulting in a sudden rise in stress levels. Conversely, when a significant number of dislocations detach from the solid solution atoms, they regain the ability to move, causing the material’s deformation resistance to return to normal and the stress level to drop rapidly. This cyclic process of pinning and detachment gives rise to the abrupt fluctuations in stress levels, creating the serrated pattern on the stress–strain curve. The serrations can be analyzed based on their location and characteristics, which provide insights into the varying internal mechanisms of DSA under different conditions [47]. By comparing the loading portion of the hysteresis loop at positive strains across various temperatures in an IF test, notable differences in serration morphology can be observed. For instance, Figure 6 illustrates these variations, highlighting how temperature affects the DSA behavior and, consequently, the shape of the serrations.

For the tests conducted at 450 °C and 550 °C, the serrations induced by dynamic strain aging (DSA) primarily exhibit characteristics of the A-type sawtooth pattern. This type of serration displays a periodic repetition, with stress drops occurring below the general level of the stress–strain curve [47,48]. As the temperature rises to 650 °C, a notable increase in the number of type B serrations is observed. Type B serrations manifest as oscillations above the general level of the stress–strain curve and are a consequence of discontinuous DSA triggered by migrating dislocations within the deformation band. As the temperature escalates, a gradual transition from A-type to B-type serrations is evident, often culminating in their coexistence, as exemplified in the 650 °C test. To assess the impact of axial and torsional loading on the DSA mechanism, constant temperature uniaxial tensile tests were performed at various temperatures. The results of these tensile tests reveal that the type of DSA undergoes a gradual transformation with increasing temperature, mirroring the trends observed in the torsional fatigue tests. Specifically, as the temperature rises, B-type DSA becomes prominent, with numerous serrations appearing on the tensile stress–strain curve immediately after yielding at 650 °C. This is a defining characteristic of B-type DSA [47,48].

**Figure 7:**
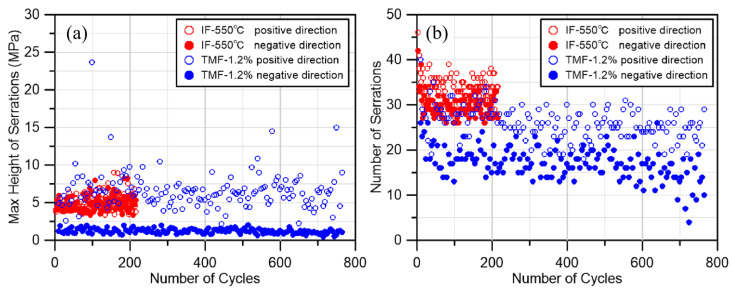
(**a**) Comparison of serrations height in TMF and IF tests under 1.2% amplitude. (**b**) Comparison of serrations number in TMF and IF tests under 1.2% amplitude.

As illustrated in Figure 7, the IF 550 °C test and the TMF 1.2% test were selected for comparison. The loading rate and strain amplitude of these two tests were identical, with the difference only being that one test was at a constant 550 °C while the other was at 350–550 °C. It can be found that under the IF test, the number of serrations appearing in the positive and negative directions is basically the same, and the maximum value of the serrations in each cycle is also basically the same, while under the TMF condition, since the negative direction is the range of the temperature decreasing, it can be seen that the number of serrations decreases significantly, and there is a significant difference in the maximum height of the serrations. This is due to the fact that the mechanism of DSA formation changes as the temperature increases. In the temperature range of 350~450 °C, the occurrence of DSA is mainly interstitial solid solution atoms and dislocations occurring pinning, and with the increase in temperature, this type of DSA will be suppressed or even completely disappeared [1,2]. According to the results of Hong`s test [49], the DSA of the 316LN material is not obvious in the range of 350 °C. When the temperature continues to rise above 450 °C, the replacement solid solution atoms (Cr, Mn, etc.) will be pinned with the movable dislocations, and the DSA phenomenon of the material will reappear and become more obvious.

### 3.2. Friction Stress and Back Stress

The back and friction stresses are reflections of the internal stress state of the material during cyclic deformation, which can well demonstrate the changes in the internal microstructure of the material. Using the Kuhlmann-Wilsorf-Laird (KWL) method proposed by Cottoler, the back and friction stresses can be obtained directly from the hysteresis loop at each cycle [50]. However, little information can be found in previous reports on the analysis of back and friction stresses in TMF tests. This is mainly due to the fact that the KWL method requires a plasticity hysteresis loop data to calculate the back and friction stresses. Usually, plasticity data can be obtained by subtracting the elastic strain from the total strain. However, the elastic modulus of a material is temperature dependent. Therefore, the continuous change in temperature under TMF conditions means that each point in a hysteresis loop has a different elastic modulus, making it difficult to obtain accurate plastic strains using the KWL method.

**Figure 8:**
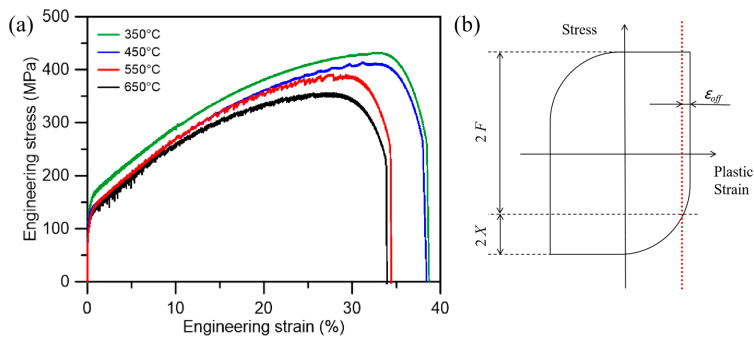
(**a**) Monotonic tensile tests at various temperature. (**b**) Schematic of KWL method for calculating friction stress and back stress.

To address this issue, the elastic modulus of 316LN stainless steel was measured at various temperatures spanning from 350 °C to 650 °C through monotonic tensile tests, as illustrated in Figure 8a. Polynomial fitting was employed to derive the variation curve of the material’s Young’s modulus with respect to temperature within the experimental range. To account for the reduction in stiffness due to damage incurred during cyclic deformation, the slope of each unloading segment (within the 85–95% strain range) was assessed and compared against the standard modulus within the respective temperature range. This comparison facilitated the determination of the damage coefficient, which in turn allowed for the calculation of the elastic modulus at all parameter points within the hysteresis loop at cyclic temperatures. For experiments conducted at constant temperatures, the slope of each unloading segment (within the 85–95% strain range) was directly measured to represent the modulus for that particular cycle. The elastic strain at each point on the hysteresis curve was computed by dividing the total stress at that point by the modulus prevalent under those conditions. Subsequently, the plastic strain at each point was obtained by subtracting the calculated elastic strain from the total strain at that corresponding point in the hysteresis loop.

**Figure 9:**
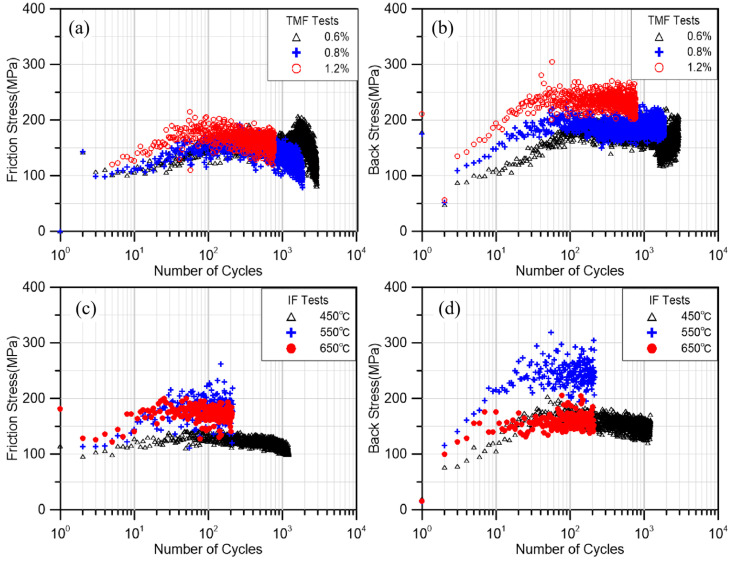
(**a**) Friction stress of TMF tests. (**b**) Back stress of TMF tests. (**c**) Friction stress of IF tests. (**d**) Back stress of IF tests.

The stresses are categorized into friction and back stresses by the KWL method shown in Figure 8b, stress was decomposed into friction stress, *F*, and back stress, *X*. The offset value *ε_off_* chosen in the KWL method to describe the yield range of the material is an uncertain covariate, which is usually chosen in a range from 10^−3^ to 10^−6^ depending on the specific data. In this study, a number of columns of the related literature were referred to as well as analyzed and debugged with specific data, and finally *ε_off_* = 10^−4^ was chosen for all the tests [51,52,53]. By writing the MATLAB program, the total stress of each loop of the hysteresis loop of the experimental results were decomposed into back stress and friction stress, and the results are shown in Figure 9.

From Figure 9, it is evident that in torsional TMF tests, the influence of amplitude on friction stress is relatively insignificant. Initially, as cyclic hardening occurs, friction stresses at various amplitudes rise to a peak before gradually declining. A notable distinction arises in the test results obtained at smaller amplitudes. Specifically, the friction stresses exhibited significant secondary hardening, mirroring the secondary growth observed in total stresses. This alignment suggests that the material’s secondary hardening is primarily driven by an increase in the friction stress component. The mechanism behind this secondary hardening, particularly at small amplitudes, can be attributed to the continuous accumulation of planar slip during deformation cycles. This accumulation leads to a secondary rise in friction stress. However, as the deformation amplitude increases, the slip pattern transitions from planar slip to cross-slip. This transition facilitates the cancelation of dissimilar dislocations on different slip surfaces, resulting in a decrease in local dislocation density and a subsequent smooth decline in friction stress [54]. Furthermore, the back stress increases with the amplitude and reaches a stable state at the end of cyclic hardening. This observation indicates that the initial hardening of the material is a combined effect of increasing back stress and friction stress. Conversely, the subsequent cyclic softening is primarily driven by the reduction in friction stress.

Under IF conditions, it can be seen that the frictional stresses at 550 °C and 650 °C are significantly higher than the test results at 450 °C. Previous studies have pointed out that there is a significant positive correlation between friction stress and DSA [3]. This suggests that a stronger DSA phenomenon occurs in the material at 550 °C and 650 °C, resulting in higher friction stresses compared to those at 450 °C. The back stress is affected by the overall deformation coordination property of the material, which should normally decrease gradually with the same load as the temperature increases and the material softens leading to enhanced deformation coordination property [55,56,57]. However, in the IF550 °C test, the back stress at 550 °C was significantly increased, which was similar to the experimental result at 450 °C. This is because the frequent and intense DSA at this temperature leads to an increase in the material dislocation density, an increase in the deformation resistance of the specimen, and a corresponding increase in the back stress. At 650 °C, in addition to the higher deformation coordination capacity due to the temperature increase, the thermal recovery effect at higher temperature is also applied. Due to the lower fault energy of the FCC layer, there is basically no cross-slip under the lower temperature condition (450 °C). With the temperature increase, the material layer fault energy increases, the number of cross-slip increases, the dislocations of different slip surfaces of different numbers eliminate each other, and the overall dislocation density of the material decreases, which leads to a significant decrease in the growth rate of the backs tress of the material, which is much lower than that of other experiments. At the same time, the lower growth in back stress also makes the overall stress response of the material at 650 °C significantly lower than that of the other two groups of tests with the same amplitude.

Comparing the stress compositions of the two loading schemes, isothermal fatigue (IF) and torsional fatigue (TMF), notable differences emerge in the percentages of back stress and friction stress. In the TMF test, a higher percentage of back stress is observed compared to the IF test. This is attributed to the inclusion of a low-temperature section in the TMF experiment, which significantly enhances the difficulty of material deformation. Consequently, a greater stress is required to coordinate local deformation, leading to an increase in back stress. In both loading schemes, the hardening and softening of the material are achieved through the combined action of back stress and friction stress. However, the relative contributions of these stress components differ between the two tests. In the IF experiment, as the temperature rises, the growth rate and absolute value of the back stress decrease. Consequently, the cyclic hardening of the material is primarily driven by the cyclic hardening of the friction stress. Similarly, during the softening stage, the dominance of friction stress is evident.

### 3.3. Microanalysis

#### 3.3.1. Surface Crack

**Figure 10:**
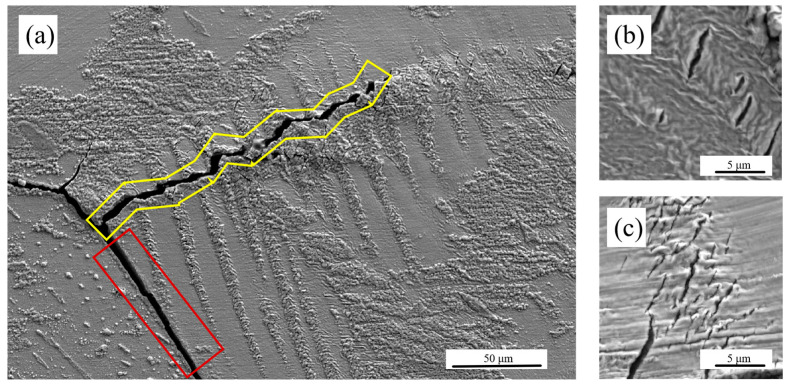
(**a**) Surface crack and oxidation distribution. (**b**) Crack in the slip bands of the TMF-0.6% test. (**c**) Crack in the slip bands of the TMF-1.2% test.

The surface oxidation and cracking of individual specimens under torsional loading have similar trends. As shown in Figure 10a, there is an obvious preferential oxidation along the slip band inside the grains, which is due to the fact that the extrusion-extrusion structure formed in the slip deformation facilitates the infiltration of oxygen elements and the oxidation reaction [58,59]. At the same time, a large number of cracks appeared inside the slip band oxides, with the direction maintaining a 45° angle with the overall loading direction. The number of cracks and the degree of crack opening inside the slip band increased significantly with the increase in the experimental strain load, as illustrated in Figure 10b,c.

The crack pattern observed in the specimens reveals a complex interplay between primary and secondary cracks, as well as intergranular and transgranular cracks. The primary cracks extended predominantly along the axial direction, while secondary cracks were present and extended in the circumferential direction. This composite crack formation indicates a multifaceted failure mechanism. The intergranular cracks, highlighted in the red area of Figure 10a, extended along grain boundaries. These cracks are often indicative of weak grain boundaries or intergranular corrosion, where the material’s strength is compromised at the grain interfaces. On the other hand, transgranular cracks, shown in the yellow area of the same figure, penetrated through the grains. Notably, these cracks did not follow the slip bands inside the grains but were perpendicular to them. When crossing slip bands, the transgranular cracks changed direction, often connecting to oblique cracks formed by oxides within the slip bands. The step-like extension route of the penetration cracks as they crossed slip bands suggests that cracks within the slip band oxides formed prior to the main crack. These pre-existing cracks influenced the direction of extension of the main crack as it progressed. This observation is crucial because it indicates that the oxides within the slip bands played a significant role in initiating and guiding crack propagation.

#### 3.3.2. Dislocation Evolution

**Figure 11:**
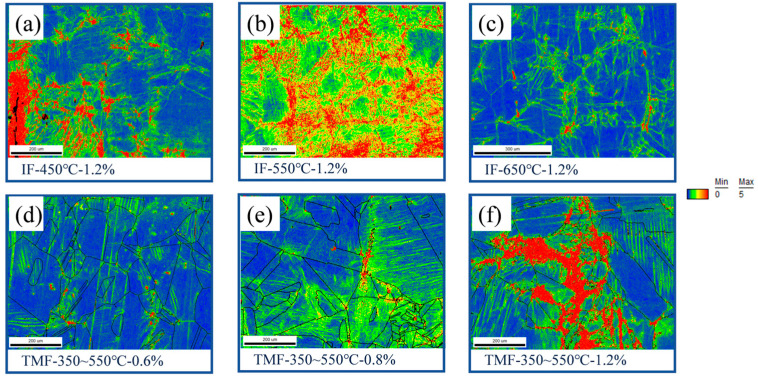
(**a**) KAM results of tests under IF-450 °C-1.2%. (**b**) KAM results of tests under IF-550 °C-1.2%. (**c**) KAM results of tests under IF-650 °C-1.2%. (**d**) KAM results of tests under TMFF-350~550 °C-0.6%. (**e**) KAM results of tests under TMFF-350~550 °C-0.8%. (**f**) KAM results of tests underTMFF-350~550 °C-1.2%. The color from blue to red in the picture indicates a gradual increase in the KAM value of the location.

The portion of the specimen directly opposite to the macroscopic crack was intercepted and a flat surface of 3 mm width was polished according to the process shown in Figure 2, which was mechanically polished as well as electrolytically polished by some means of treatments to characterize it by EBSD. The KAM map was calculated by EBSD mapping, as shown in Figure 11.

Upon comparison, it is observed that an increase in strain amplitude during the TMF test leads to enhanced material deformation. Notably, more pronounced concentrated deformation tends to occur along grain boundaries, as indicated by the reddish regions in the KAM diagram. In contrast, during the IF test at 450 °C, the stress concentration patterns resemble those observed in the TMF test with a 1.2% strain amplitude. The deformation is predominantly localized at grain boundaries and micro-cracks, with minimal internal deformation within the grains, as evidenced by the blue-green regions in the KAM diagram. Conversely, the results of the IF test at 550 °C exhibit marked differences. A significant degree of deformation and stress concentration is also observed within the grains, appearing as an overall yellow hue in the KAM diagram. At 650 °C, the material demonstrates reduced deformation. Both internal grain deformation and grain boundary stress concentrations are minimal, with very few large deformations highlighted in red on the KAM diagram.

Typically, KAM results provide insight into the distribution of dislocations [60]. A higher KAM value, represented by a redder hue on the graph, signifies a greater dislocation density at that specific location. In the context of the IF test conducted at 550 °C, dynamic strain aging (DSA) effects were most pronounced, resulting in a significantly higher dislocation density within the crystal compared to other experimental conditions. When the temperature rises to 650 °C, numerous studies have demonstrated that 316LN stainless steel undergoes considerable thermal recovery [54,60]. As the temperature increases, the stacking fault energy of austenitic materials also rises. At higher stacking fault energies, expanding dislocations are more prone to clustering and forming screw dislocations, which are more likely to propagate through cross-slip mechanisms. During cross-slip, dislocations with opposite signs annihilate each other, leading to a substantial reduction in the dislocation density within the material. A lower dislocation density translates to a reduced stress response, particularly the back stress primarily governed by short-range dislocation stress. This observation aligns with the results from previous stress decomposition analyses of IF tests conducted at various temperatures.

In the context of DSA behavior, the mutual pinning effect between solid solution atoms and movable dislocations obstructs the transition of dislocations to different slip planes. Essentially, DSA inhibits the occurrence of cross-slip. Nevertheless, the sliding mode of dislocations is also contingent upon the extent of deformation. When the plasticity reaches a sufficient level, the initiation of climb mechanisms across multiple slip planes and dislocations occurs. This process suppresses the planar slip that is predominantly influenced by DSA, thereby making cross-slip the dominant mechanism. When dislocations undergo cross-slip on various slip planes, they encounter and annihilate each other based on their Burgers vectors, leading to a reduction in dislocation density and promoting rearrangement and combination within the entanglement of dislocations. Consequently, DSA and cross-slip can be considered as two competing mechanisms to a certain extent.

**Figure 12:**
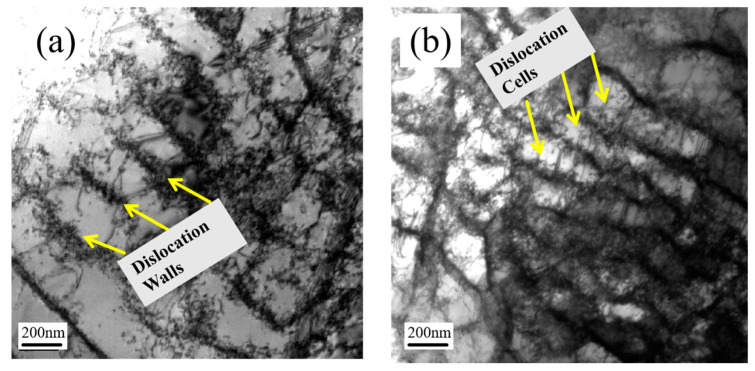
(**a**) TEM mapping result of IF test at 550 °C. (**b**) TEM mapping result of IF test at 650 °C.

Under the combined effect of different combinations of temperature and strain conditions, the material tends to exhibit different dislocation states internally. The specimens at 550 °C and 650 °C were subjected to TEM characterization and the mapping result is shown in Figure 12.

As depicted in Figure 12, the material at 550 °C exhibits a complex slip pattern. Both slip band structures characteristic of planar slip and dislocation walls indicative of cross-slip can be observed. The presence of cross-slip contributes to a lower back stress at 550 °C compared to the IF test conducted at 450 °C. The abundance of planar slip confirms that DSA is active at 550 °C. As the temperature increases to 650 °C, thermal recovery becomes dominant in austenitic stainless steel, with cross-slip taking precedence. Within the material, numerous intact dislocation cell structures form. These abundant wave slip structures consume the free dislocations, thereby inhibiting further DSA activity. Consequently, the back stress at 650 °C is notably lower than in other tests, and the increase in friction stress is ultimately less than the results obtained from tests at 550 °C. Similarly, the ongoing thermal recovery process leads to a decrease in dislocation density within the material, which aligns with the KAM results derived from the EBSD mapping.

## 4. Conclusions

This study examined the effects of temperature and mechanical loading on the mechanical response to the torsional fatigue and microstructure evolution of materials through IF tests at various temperatures and TMF tests at different strain amplitudes under torsional loading conditions. Utilizing the KWL method and various microstructure characterization techniques, the following conclusions were drawn:

1. Temperature significantly impacts DSA behavior in materials. Similar to axial loading tests, DSA behavior in 316LN material is most pronounced during torsional loading at 550 °C.
2. Cracks formed in the oxide layer within slip bands on the specimen surface precede the main cracks and influence their propagation direction during the crack growth process.
3. Under conditions of small deformation, secondary hardening occurs in the specimen due to the increased frictional stress caused by accumulated planar slip. However, this phenomenon diminishes as the strain amplitude increases.
4. Under conditions of large plastic deformation, 316LN exhibits a mixed slip pattern at 550 °C, whereas cross-slip dominates at 650 °C. This results in lower back stress and frictional stress at 650 °C compared to 550 °C.
5. The material exhibits a higher dislocation density during the 550 °C IF test due to frequent DSA. In contrast, the material has a lower dislocation density at 650 °C due to sufficient thermal recovery.

Based on ongoing research, further multiaxial TMF tests are planned across diverse temperature ranges and strain amplitudes. The objective is to explore the damage coupling mechanism under various loading directions by analyzing the discrepancies in fatigue life and failure mechanisms observed in specimens subjected to multiaxial, axial, and torsional loading. The ultimate aim is to establish a life model suitable for multiaxial TMF behavior.

## Figures and Tables

**Table 1:** Chemical composition of the as-received 316LN stainless steel (in wt%).

C	Si	N	P	Mn	Cr	Ni	Co	Mo	Cu	Nb	Fe
0.018	0.22	0.112	0.023	1.42	17.20	12.95	0.018	2.19	0.033	0.068	Bal.

## Data Availability

The original contributions presented in the study are included in the article; further inquiries can be directed to the corresponding author.

## Abbreviations

DSA	Dynamic strain aging
EBSD	Electron back scatter diffraction
EDS	Energy dispersive spectrometer
IF	Isothermal fatigue
KAM	Kernel average misorientation
KWL	Kuhlmann–Wilsorf–Laird
OPS	Oxide polishing suspension
PWR	Pressurized water reactor
SEM	Scanning electron microscope
TEM	Transmission electron microscope
TMF	Thermomechanical fatigue
WEDM	Wire electrical discharge machining
